# Genetic variation and recombination analysis of PRRSV-2 *GP3* gene in China from 1996 to 2023

**DOI:** 10.3389/fmicb.2024.1435373

**Published:** 2024-08-16

**Authors:** Chen Lv, Yajie Zheng, Kexin Liu, Gan Li, Qin Luo, Hang Zhang, Huiyang Sha, Ruining Wang, Weili Kong, Mengmeng Zhao

**Affiliations:** ^1^Guangdong Provincial Key Laboratory of Animal Molecular Design and Precise Breeding, School of Life Science and Engineering, Foshan University, Foshan, China; ^2^Henan University of Animal Husbandry and Economy, Zhengzhou, Henan, China; ^3^University of California, San Francisco, San Francisco, CA, United States

**Keywords:** PRRSV, GP3 gene, genetic variation, genetic recombination, phylogeny

## Abstract

Porcine reproductive and respiratory syndrome virus (PRRSV) has become widespread in China particularly the highly pathogenic porcine reproductive and respiratory syndromes (HP-PRRSV), NADC30, and NADC34 strains, and has posed a threat to the swine industry for over 20 years. To monitor genetic variation in PRRSV-2 GP3 strains in China, we analyzed 618 strains isolated between 1996 to 2023 and constructed phylogenetic trees. Additionally, 60 selected strains were used to analyze nucleotide and amino acid homology. PRRSV *GP3* gene exhibited nucleotide identity ranging from 78.2% to 100.0% and amino acid similarity ranging from 74.9% to 99.6%. The *GP3* gene in the 60 selected strains consisted of 254 amino acids, and amino acid mutations in the strains primarily occurred in B-cell epitopes, T-cell epitopes, and highly variable regions. The glycosylation sites of the strains used for amino acid sequence comparisons remained unaltered, except for the N^29^ site in the GD20220303-2022 strain. PRRSV-2 strains in China belong to lineages 1, 3, 5, and 8. Recombination analysis detected two recombination events, involving lineages 1 and 8. In conclusion, this study investigated multiple strains of the PRRSV-2 GP3 gene to explore the prevalence and genetic diversity of the GP3 gene in China from a gene family perspective. The results of the analyses provide a basis for clinical prevention strategies and vaccine development.

## 1 Introduction

Porcine reproductive and respiratory syndrome (PRRS) is an acute and virulent infectious disease caused by the porcine reproductive and respiratory syndrome virus (PRRSV) (Yu et al., 2022). The typical symptom of PRRS is the cyanosis of the skin at the end of the ears, commonly known as the “blue-ear disease.” Pregnant sows and piglets are the most susceptible to this virus. The symptoms in pregnant sows after infection are complicated and manifest primarily as reproductive disorders, abortion, weak fetuses, still-births, and mummified fetuses, accompanied by immunosuppressive diseases and certain respiratory diseases. In piglets, the syndrome is manifested as congenital dysplasia, hyperthermia, and interstitial pneumonitis. PRRS was initially discovered in the United States in 1986 and was called a “mystery disease”. Subsequently, it spread rapidly worldwide. This disease was initially reported in China in 1996 and subsequently spread widely and mutated into highly pathogenic strains (HP-PRRSV) in 2006 and NADC-like strains in 2012 (Snijder and Meulenberg, 1998; Gao et al., 2017; Zhang et al., 2018a; Xie et al., 2020; Liu et al., 2021; Xu et al., 2022). PRRS is highly contagious with a high mortality rate, causing huge economic losses to large-scale piggeries worldwide, and is considered one of the most important infectious diseases threatening the sustained and healthy development of the global animal breeding industry, together with various virulent infectious diseases of animals (Allende et al., 1999; Tian et al., 2007; Zhou et al., 2018; Cai et al., 2022; Liang et al., 2022; Goatley et al., 2023).

PRRSV is classified within the family *Arterivirus*, under the order *Nidoviridae* (Cavanagh, 1997). Other viruses in this family include equine arteritis virus (EAV), lactate dehydrogenase-elevating virus (LDV), and simian hemorrhagic fever virus (SHFV) (Shi et al., 2020). PRRSV is a single-stranded, positive-stranded RNA virus with spherical particles, a capsid, a smooth, ciliated surface, and a diameter of approximately 50–60 nm. The nucleocapsid is cubic in shape and has a diameter of 25–35 nm. The virus is temperature-sensitive and can be inactivated by heating to 56°C for 20 min. Organic chemicals can also destroy the virus, and chloroform, ether, and trichloromethane can inhibit its reproduction by destroying its capsid (Tong et al., 2007). PRRSV can be classified into two genotypes, namely, type 1 (European) and type 2 (American). The two genome sequences are similar by approximately about 54–60% (Meng et al., 1995), and the prevalent strains in Europe are mostly PRRSV-1 (symbolized by LV strains extracted from the Netherlands). In contrast, in China, PRRSV strains are mainly PRRSV-2 (symbolized by the VR2332 strain from the United States). Per the recent classification scheme of the International Committee for the Classification of Viruses (ICVC), PRRSV can be categorized into *Betaarterivirus suid 1* (former PRRSV-1 type) and *Betaarterivirus suid 2* (former PRRSV-2 type) under *Betaarterivirus* (Adams et al., 2017).

The genome of PRRSV spans approximately 15 kb, including at least 11 open reading frames (ORFs): ORF1a, ORF1b, ORF2a, ORF2b, ORF3-7, ORF5a, and TF. TF overlaps with the coding region of non-structural protein 2 (NSP2) of ORF1a (Fang et al., 2012). PRRSV encodes a structural protein (SP) and a non-structural protein (NSP), which can be classified into two types: major structural proteins (GP5, M, and N) and minor structural proteins (GP2, GP2a, GP3, GP4 and E) (Ke and Yoo, 2017). The remaining are non-structural proteins that constitute approximately about 75% of the genome (Ma et al., 2021). ORF1a and ORF1b are responsible for encoding two important viral replicase proteins (pp1a and pp1ab), which can be translated into 16 non-structural proteins (NSPs): NSP1α, NSP1β, NSP2, NSP2TF, NSP2N, NSP3-6, NSP7α, NSP7β and NSP8-12 (Sun et al., 2022). ORF2-7 are responsible for encoding GP2, E, GP3, GP4, GP5a, GP5, M and N (Snijder et al., 1999). These proteins play an important role in activating replicate, shear, and protease activities during replication, transcription, and translation, and some NSPs play important roles in PRRSV infection, anti-body production, and immune escape regulation (Oleksiewicz et al., 2001).

GP3, a protein encoded by ORF3, has a molecular mass of approximately 27-29 kDa and is composed of a segmented signal peptide, a domain with extensive glycosylation, a brief hydrophobic segment, and a C-terminal structural domain that is not glycosylated and varies greatly. GP3 is the most glycosylated protein in PRRSV, with seven highly conserved glycosylation sites, and is the second most conserved structural protein after GP5. Differences in antigenic epitopes and the secondary structures of GP3 sequences between different typologies and strains may be responsible for this phenomenon. GP3 also has high immunogenicity and antigenicity, which can induce neutralizing antibody production and interact with CD163, a key receptor for PRRSV entry into the cell, to increase infectivity (Vu et al., 2011; Zhang et al., 2020). GP3 is found in relatively small amounts of viral particles. It is mainly produced in cellular secretions and may act as a decoy to disperse antibodies to keep susceptible cells away from viral particles (Zhang et al., 2018b). Antibodies produced against GP3 have been experimentally demonstrated to prevent viral infections, but the ability of GP3 to generate antibodies is not as strong as that of GP5 (Guarino et al., 1999). Furthermore, the fusion of heat shock protein 70 with GP3 of PRRSV induced the production of interferon-gamma (IFN-γ) and interleukin-4 (IL-4) in porcine sera (Du et al., 2012). IL-4 production in the serum enhances the body’s immune response, protects swine, and reduces the infection rate of PRRSV (Du et al., 2012). The high degree of GP3 glycosylation may influence viral immune evasion by the virus, GP3 can also form a heterotrimer with GP2a and GP4 to dope the virion, and viral particles deficient in these three proteins lose their infectivity (Murtaugh et al., 1995; Wieringa et al., 2002; Vu et al., 2011).

## 2 Materials and methods

### 2.1 Data collection

A comprehensive genetic variation analysis of the *GP3* gene in Chinese PRRSV-2 was conducted by selectively screening 618 PRRSV-2 strains (610 isolates from China and 8 classical strains from the United States) from the GenBank database available on the NCBI website^1^ (Supplementary Table 1). The study included a selection of strains from various years and lineages (lineages 1, 3, 5, and 8). A total of 60 representative strains with GP3 sequences were meticulously chosen from a larger pool of 618 strains, including representatives from various sources such as representative, vaccine, and prevalent strains. This thorough selection process was carried out to ensure the inclusiveness and comprehensiveness of the genetic variation analysis of the *GP3* gene in Chinese PRRSV-2 (Table 1).

**TABLE 1:** The GP3 reference sequences of 60 PRRS strains.

Year	Area	Strain	Genbank accession number
1993	USA	ATCC VR-2332	U87392
1996	China	CH-1a	AY032626
1998	USA	RespPRRS MLV	AF066183
1999	USA	MLV RespPRRS Repro	AF159149
2000	China	BJ-4	AF331831
2005	USA	MN184A	DQ176019
2005	USA	MN184B	DQ176020
2006	China	R98	DQ355796
2006	China	CC-1	EF153486
2006	China	JXA1	EF112445
2006	China	HUN4	EF635006
2006	China	TJ	EU860248
2006	China	HUB1	EF075945
2006	China	HUB2	EF112446
2006	China	JX143	EU708726
2006	China	S1	DQ459471
2007	USA	MN184C	EF488739
2007	China	DY	JN864948
2007	China	SHH	EU106888
2007	China	GD	EU109503
2007	China	Henan-1	EU200962
2007	China	BJ	EU825723
2007	China	XH-GD	EU624117
2008	USA	NADC30	JN654459
2008	USA	NADC31	JN660150
2008	China	PRRSV01	FJ175687
2008	China	PRRSV02	FJ175688
2008	China	PRRSV03	FJ175689
2008	China	CH-1R	EU807840
2008	China	JXA1 P80	FJ548853
2008	China	WUH3	HM853673
2009	China	GS2002	EU880441
2009	China	GS2003	EU880442
2009	China	GS2004	EU880443
2009	China	CH2002	EU880438
2009	China	CH2003	EU880440
2009	China	CH2004	EU880439
2009	China	JXA1-P120	KC422727
2009	China	ZP-1	HM016159
2009	China	SX-1	GQ857656
2010	China	QY2010	JQ743666
2010	China	JX	JX317649
2011	China	GM2	JN662424
2011	China	QYYZ	JQ308798
2011	China	YN-2011	JX857698
2013	China	JL580	KR706343
2014	China	CHsx1401	KP861625
2014	China	FJ1402	KX169191
2014	China	FJM4	KY412888
2015	China	HNyc15	KT945018
2015	China	HNjz15	KT945017
2015	China	SD-A19	MF375260
2015	China	GDsg	KX621003
2017	China	SD17-36	MH121061
2018	China	SCya18	MK144543
2019	China	HNLCL15-1903	ON462043
2020	China	rJXA1-R	MT163314
2021	China	ZJqz21	OK274266
2022	China	GD20220303	OQ459668
2023	China	YN-DZ	OQ944349

### 2.2 PRRSV-2 *GP3* gene sequence analysis

GP3 nucleotide homology was examined using the Clustal W tool within the MegaAlign feature of DNAStar software (version 7.0, Madison, WI, USA), and the nucleotide sequences were then translated into the corresponding amino acid sequences using the EditSeq program to analyze GP3 amino acid homology. Amino acid sequences were analyzed using the DNAStar and MEGA software (version 5.1, MegaLimited, Auckland, New Zealand).

### 2.3 Phylogenetic analysis

Phylogenetic analysis of the 618 PRRSV strains was performed using the GP3 sequences. Phylogenetic trees were constructed using the maximum likelihood (ML) and neighbor-joining (NJ) methods in the MEGA software with 1000 times self-expansion analyses and default settings for other parameters. The obtained phylogenetic tree results were imported into the online visualization and editing software The Interactive Tree of Life^2^ for annotation and editing.

### 2.4 Recombination analysis

The RDP4 software employs seven distinct algorithms (RDP, GENECONV, BootScan, MaxChi, Chimera, SiScan, and 3Seq) to forecast possible recombination occurrences. Strains whose analyses were positive (+) for more than four algorithms with *p* < 0.05 were considered recombinant, assuming that recombination events had occurred. Strains identified as having undergone recombination were validated using SimPlot software (version 3.5.1).

## 3 Results

### 3.1 Nucleotide and amino acid homology analysis of *GP3* gene

To investigate genetic variation in the PRRSV *GP3* gene, 60 strains from lineages 1, 3, 5, and 8 were selected. Nucleotide and amino acid homology were analyzed using DNAStar software to fully comprehend the genetic evolutionary relationships among these different lineages. The findings indicated that the nucleotide similarity among the *GP3* genes of the selected strains varied between 78.2 to 100% whereas the amino acid similarity ranged from 74.9 to 99.6%.

The lowest nucleotide homology (78.2%) was found in GD20220303-2022 and R98-2006 strains, while strains GD20220303-2022 and R98-2006 had the lowest amino acid homology (74.9%). The highest nucleotide homology (100%) was observed between the following set of strains: ZJqz21-2021 and NADC30-2008, YN-2011-2011 and BJ-4-2000, GS2002-2009, GS2003-2009 and GS2004-2009, YN-2011-2011, BJ-4-2000 and MLV RespPRRS Repro-1999, S1-2006 and PRRSV01-2008, ATCC VR-2332-1993 and PRRSV03-2008, CH2002-2009, CH2003-2009 and CH2004-2009, rJXA1-R-2020 and JXA1 P80-2008, YN-DZ-2023 and JXA1-2006, QY2010-2010 and QYYZ-2011 strains. Additionally, the amino acid similarity between strains NADC30-2008 and ZJqz21-2021, BJ-4-2000 and MLV RespPRRS Repro-1999, S1-2006 and PRRSV01-2008, ATCC VR-2332-1993 and PRRSV03-2008, GS2002-2009, GS2003-2009 and GS2004-2009, BJ-4-2000, MLV RespPRRS Repro-1999 and YN-2011-2011, TJ-2006 and BJ-2007, CH2002-2009, CH2003-2009 and CH2004-2009, QY2010-2010 and QYYZ-2011 were 99.6% (Table 2).

**TABLE 2:** Nucleotide and amino acid homology analysis of *GP3* gene (%).

Lineage		1	3	5	8
1	nt	90.5–100^B^	83.1–86.1	78.2^A^–86.2	80.5–86.3
	aa	88.6–99.6^B^	82.4–86.3	74.9^A^–85.5	78.4–85.5
3	nt		88.8–98.7	83.7–90.6	85.4–91.2
	aa		89.4–97.6	83.9–90.6	83.1–89.0
5	nt			83.4–100^B^	82.6–91.6
	aa			80.4–99.6	77.3–89.4
8	nt				85.6–100^B^
	aa				81.6–99.6

^A^Indicates the lowest homology.

^B^Indicates the highest homology.

### 3.2 Comparison of GP3 amino acid sequence

**FIGURE 1:**
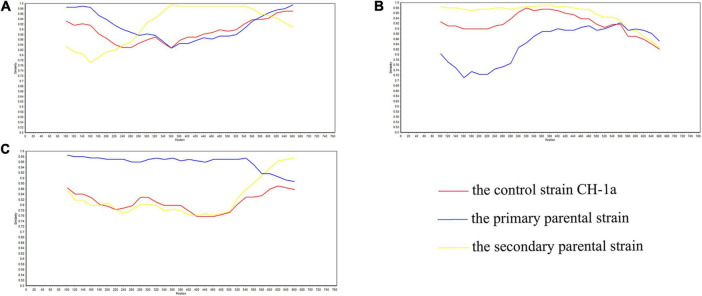
SimPlot validation of the recombination events in the *GP3* gene. **(A)** Virulent strain GDDX-2018-2018 in which a recombination event occurred. **(B)** Virulent strain FJ1402-2014 in which a recombination event occurred. **(C)** Virulent strain CH-SCMY-2-2019-2019 in which a recombination event occurred. The blue line represents the primary parental strain, the yellow line represents the secondary parental strain, and the red line represents the control strain CH-1a.

MegAlign feature in DNAStar software was utilized to analyze the 60 amino acid mutation sites in PRRSV GP3. Across all 60 strains selected for sequence comparison, the GP3 protein consistently had 254 amino acids. Notably, mutations were primarily concentrated around residues 2–32 and 200–230, encompassing the B-cell epitope region, T-cell epitope region, and high variant region (HVR). It is worth mentioning that the GD20220303 strain exhibited an N^29^ → D^29^ mutation at the glycosylation site of amino acid N^29^, while the remaining 59 strains showed no mutations in the seven glycosylation sites (N^29^, N^42^, N^50^, N^131^, N^152^, N^160^, and N^195^). The 7 strains of lineage 1 showed the highest concentration of mutations at positions 2-32 compared with those of lineages 3, 5, and 8, and the 19 strains of lineage 5 showed the lowest concentration of mutations compared with those of lineages 1, 3, and 8. All 60 strains showed the F^32^ → Y^32^ mutation at position 32, with the lowest concentration of mutations at positions 110–130 and 170–200; the highest concentration of mutations was found in the epitope region of the B-cells. The B-cell epitope region exhibited the highest variation. Some strains had characteristic mutations according to the lineage, such as T^6^ → A^6^, V^25^ → A^25^, and S^83^ → E^83^ in lineage 1, R^71^ → G^71^ in lineages 5, and R^71^ → K^71^, L^73^ → F^73^, and Q^214^ → R^214^ and 8 (Figure 1).

### 3.3 Phylogenetic analysis

**FIGURE 2:**
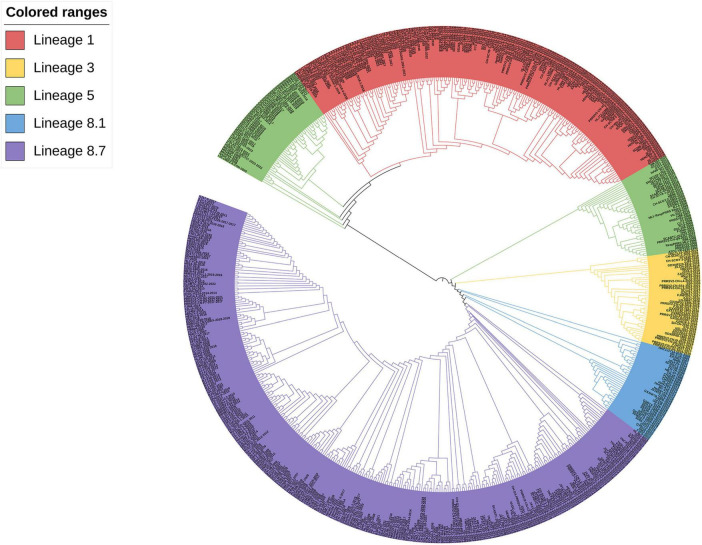
Phylogenetic analysis of the PRRSV *GP3* gene of 618 strains. The phylogenetic tree was constructed by the ML method using MEGA software (version 5.1, MegaLimited, Auckland, New Zealand), and self-expansion analysis was performed 1000 times.

**FIGURE 3:**
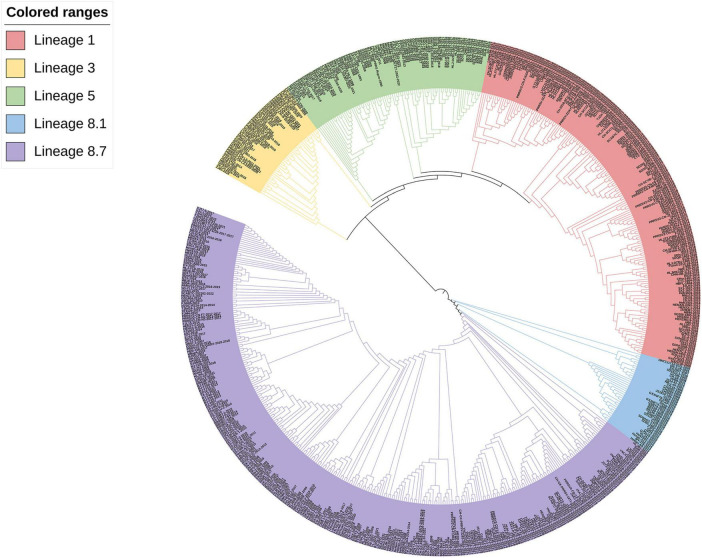
Phylogenetic analysis of the PRRSV *GP3* gene of 618 strains. The phylogenetic tree was constructed by the NJ method using MEGA software (version 5.1, MegaLimited, Auckland, New Zealand), and self-expansion analysis was performed 1000 times.

According to the worldwide PRRSV categorization framework, 618 PRRSV-2 strains were identified from the NCBI website. Analysis of GP3 sequences sourced from the GenBank database revealed that the common PRRSV-2 strains in China belong to lineages 1, 3, 5, and 8, with lineage 8 being closer to lineages 3 and 5 and farthest from lineage 1 by the ML method, but lineage 8 is more closely related to lineage 1 and more distantly related to lineages 3 and 5 by the NJ method. The recent dominant strains and mostly newly emerged strains in the last three years belong to lineages 1, 5, and 8 (Figures 2, 3).

### 3.4 Recombination analysis

**FIGURE 4:**
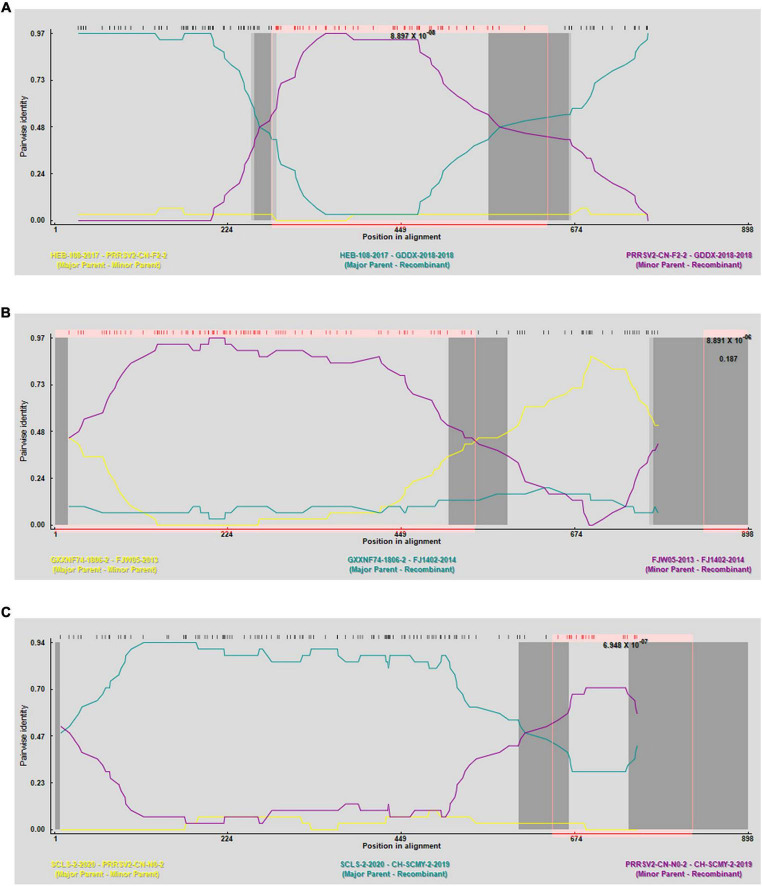
Prediction of genetic recombination events in GP3 using RDP4 software. **(A)** Strain GDDX-2018-2018 in which recombination events occurred. **(B)** Strain FJ1402-2014 in which recombination events occurred. **(C)** Strain CH-SCMY-2-2019-2019 in which recombination events occurred (R, RDP, G, GENECONV, B, BootScan, M, MaxChi, C, Chimaera, S, SiScan, T, 3Seq).

**FIGURE 5:**
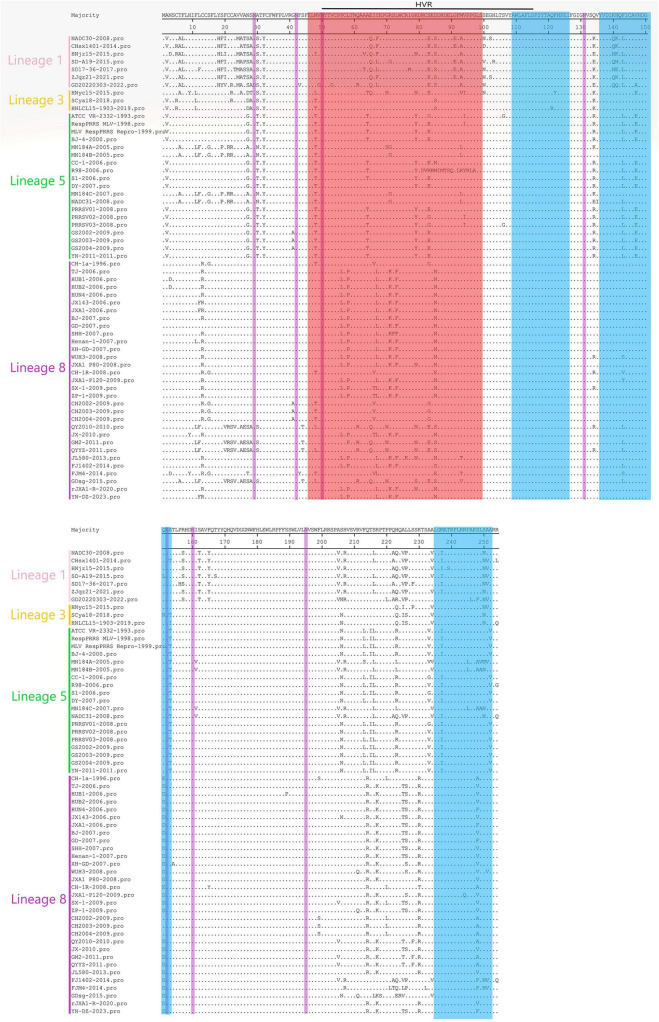
Amino acid sequences of the 60 PRRSV GP3 strains were compared with each other in lineages 1, 3, 5, and 8, with the red region representing B-cell epitopes, the blue region representing T-cell epitopes, the purple region representing glycosylation sites, and the green region representing amino acid sequences inserted.

The RDP4 software was employed to predict potential recombination occurrences, which were then confirmed using SimPlot. Three possible recombination events were identified in GP3, two of which were verified by SimPlot with *p* < 0.05, proving that the results were statistically significant (Figure 4). Event A occurred in lineages 3 and 8, indicating recombination between lineage 3 and 8, events C occurred in lineages 1 and 3, indicating a recombination event between these two lineages 1 and 3; and event B occurred exclusively in lineage 8, suggesting recombination within lineage 8 (Table 3). RDP4 detected recombination in the CH-SCMY-2-2019-2019 and GDDX-2018-2018 strains belonging to lineages 1 and 8 respectively. The recombinant GDDX-2018-2018 strain had HEB-108-2017 strain as the primary parental strain, and PRRSV2-CN-F2-2019-2019 as the secondary parental strain. For the CH-SCMY-2-2019-2019 strain, the primary and secondary parental strains were SCLS-2-2020 and PRRSV2-CN-N0-2021-2021, respectively; SimPlot confirmed the occurrence of these two recombination events (Figure 5).

**TABLE 3:** Recombination analysis of the *GP3* gene.

Recombination event	Recombinant strain (lineage)	Main parental strain (lineage)	Minor parental strain (lineage)	Recombinant breakpoint	Recombination analysis method
1	GDDX-2018-2018(8)	HEB-108-2017(8)	PRRSV2-CN-F2-2019-2019(3)	244–277 (544–649)	RDP (*P* = NS) GENECONV (*P* = 1.353 × 10^–5^) BootScan (*P* = 9.942 × 10^–8^) MaxChi (*P* = 1.911 × 10^–8^) Chimaera (*P* = 9.894 × 10^–9^) SiScan (*P* = 1.554 × 10^–10^) 3seq (*P* = 3.147 × 10^–18^)
2	FJ1402-2014(8)	GXXNF74-1806-2018(8)	FJW05-2013(8)	18–745 (493–569)	RDP (*P* = 7.424 × 10^–3^) GENECONV (*P* = 2.606 × 10^–2^) BootScan (*P* = NS) MaxChi (*P* = 3.519 × 10^–4^) Chimaera (*P* = NS) SiScan (*P* = 3.457 × 10^–10^) 3seq (*P* = 4.089 × 10^–3^)
3	CH-SCMY-2-2019-2019(1)	SCLS-2-2020(1)	PRRSV2-CN-N0-2021-2021(3)	583–646 (7–718)	RDP (*P* = 6.948 × 10^–7^) GENECONV (*P* = 1.804 × 10^–5^) BootScan (*P* = 2.082 × 10^–3^) MaxChi (*P* = NS) Chimaera (*P* = 4.948 × 10^–2^) SiScan (*P* = 1.214 × 10^–3^) 3seq (*P* = 3.129 × 10^–5^)

## 4 Discussion

Following the initial identification of PRRSV in China in 1996, continuous mutation and genetic recombination events have resulted in its extensive proliferation, presenting a significant hurdle for the swine farming sector. GP3, with its high immunogenicity and antigenicity, plays an important role in PRRSV infection and the production of neutralizing antibodies (Wang et al., 2014). To study more deeply the variation and genetic evolution of the PRRSV-2 *GP3* gene in China, we analyzed the nucleotide and amino acid homology of 60 representative strains from different lineages, as well as their amino acid sequence comparisons. Our findings showed that the nucleotide homology of GP3 ranged from 78.2% to 100.0%, while the amino acid homology ranged from 74.9 to 99.6%. The Chinese classical CH-1a strain had the highest nucleotide and amino acid homology with the MLV RespPRRS Repro strain compared to the eight classical strains from the United States, with 91.6% and 88.6%, respectively, and also had high homology with the newly emerged YN-DZ strain from China in 2023, with 95.8% of the nucleotides and 92.5% of the amino acids, respectively 92.5%. Through the homology analysis of these 60 strains (Table 2), it was observed that lineage 5 strains displayed the most significant differences in nucleotide and amino acid homology. This suggests notable recombination and variation within lineage 5, indicating the presence of genetic mutations in the *GP3* gene. Lineage 3 strains emerged later in China, with a lower clinical detection rate. In contrast, lineage 5 strains, represented by strains such as BJ-4, had a comparatively longer period of circulation in China than lineage 3. However, lineage 5 strains exhibited weaker infectivity in natural environments, resulting in a lower clinical detection rate (Guo et al., 2018). Lineage 8 strains, represented by strains such as CH-1a, were the earliest to emerge and have become prevalent in China. Furthermore, lineage 8 strains were further classified into sublineages 8.1, 8.7, and 8.9. In contrast, strains from lineages 1, 3, and 5 became more prevalent in China later than those from lineage 8. Most strains detected in China belong to lineage 8, making it the dominant circulating lineage in the country.

Amino acid deletions, insertions, and substitutions are the key factors affecting mutations in PRRSV. In this study, a sequence comparison of amino acids was performed, revealing several mutations in the PRRSV-2 type GP3 protein. These mutations mainly occurred in the B-cell epitope region, the T-cell epitope region, and the HVR. Although all strains across the spectrum exhibited varying degrees of mutations in these three regions, none of them showed any additions or deletions. The GP3 protein is known to be the most heavily glycosylated protein in PRRSV. Among the 60 strains analyzed, only the first glycosylation site of the GD20220303 strain had a mutation from N^29^ to D^29^, while the remaining six glycosylation sites and all glycosylation sites of the other 59 strains remained unmutated. This suggests a high degree of conservation in the glycosylation sites of GP3 across different PRRSV subtypes. Studies have demonstrated functional similarities between the glycosylation sites on GP3 and GP5 proteins, highlighting their crucial role in PRRSV immune evasion (Liu et al., 2011), therefore, further research on these glycosylation sites is required.

Phylogenetic analysis revealed that Chinese native PRRSV-2 strains belong to lineages 1, 3, 5, and 8, with lineages 1 and 8 exhibiting a more pronounced pattern of occurrence. In the constructed phylogenetic tree, strain CH-1a was most closely homologous to HB-1-sh strain from China and shared lineage 8 with the YN-DZ strain; it was most closely homologous to ATCC VR-2332 strain in lineage 5 from the United States. The genetic variations: between the *GP3* and the *GP5* (GP5a, which is prone to variation among structural proteins) genes, as well as among the *NSP2* gene, which undergoes frequent high-level variations and is susceptible to deletions, were compared (Luo et al., 2023a,b; Zhang et al., 2023). In the evolutionary tree generated by the ML method for GP3, lineages 1 and 5 showed a closer relationship, as did lineages 3 and 8. Lineage 5 could be divided into two parts, one part of the strain was located in the same branch as that of lineage 1 and is further away from lineage 8, and the other part of the strain is closer to lineage 3, but both parts are closer to each other than to the other lineages and to both lineages 1. Strains in lineage 3 were closer to lineage 8, suggesting that recombination might have occurred in lineages 3 and 8, a possibility that was also successfully predicted by RDP4 and identified by SimPlot. In contrast, the NJ evolutionary tree indicated that lineages 1 and 5 were closely related, lineages 1 and 3 were more distantly related, and lineages 3 and 8 were genetically distant from each other. Taken together, it can be inferred that the evolutionary tree constructed using the ML method had the highest degree of conformity with the actual situation. In addition, lineages 1 and 8 had a greater predominance in the prevalence of PRRS and have been the most prevalent lineages in China in recent years, which is consistent with the results of the two studies mentioned above (Zhang et al., 2022, 2023; Luo et al., 2023a). Variations in the phylogenetic tree could be linked to the quantity, timing, and diversity of the selected aggressive strains. In this study, we posited that variances in genetic distances could stem from factors such as the number of aggressive strain samples, duration, specific genes, and targeted algorithms, along with the genes and algorithms used for target analysis. In the last three years, most PRRSV-2 strains belonged to lineages 1, 5, and 8. 5 of the 11 new strains that emerged in 2023 belonged to lineage 1 along with the currently prevalent NADC30-like and NADC34-like strains in China, and the numbers of strains belonging to lineages 5 and 8 were 4 and 2, respectively, with no strains belonging to lineage 3, which is the same as the current predominance of PRRSV in China. This is the same as the current predominant PRRSV lineage 1 and 8 results in China. Based on our findings, we hypothesize that the prevalence trend of lineage 1 may continue to expand in the future and become the most prevalent lineage in China (Tian et al., 2007).

Since its identification in China, PRRSV has experienced a swift evolutionary process and has swiftly proliferated across the nation. Initial identification of the PRRSV NADC30 strain occurred in 2008 in the United States. At the end of 2012, the discovery of HENAN-XINX, HNyc15, JL580, and HENAN-HEB strains, which are homology to the NADC30 strain, was reported in China; these were named NADC30-like (NADC30-like) strains, which rapidly spread to several regions of the country and formed independent branches with an increased percentage of clinical detection (Tian, 2017). NADC30-like strains are less pathogenic than HP-PRRSV strains and are prone to recombination with classical strains such as CH-1a and HP-PRRSV strains (Zhou et al., 2018). In 2015, the combined presence of NADC30-like and HP-PRRSV strains increased becoming leading strains in China. In 2017, NADC34-like strains were initially identified in Liaoning Province, and by 2021, NADC34-like strains were reported in several other Chinese provinces (Xu et al., 2022). Reports suggest that NADC34-like strains may emerge as the leading PRRSV epidemic strains in China (Zhao et al., 2022). PRRSV undergoes mutations and recombination, resulting in more complex PRRSV strain types of higher virulence, making PRRS prevention, control, and decontamination more difficult. Natural genetic recombination between strains used in vaccines and those in the wild is possible, and recombination events of the GM2 strain can occur between the recombinant vaccine strain MLV RespPRRS Repro and the wild strain QYYZ, suggesting that recombination events are key to the evolution of PRRSV (Liu et al., 2021).

In this study, three sets of recombination events were identified by performing recombination analysis, and the presence of two of these sets was verified using the SimPlot software. Lineages 1, 3, and 8 had a higher likelihood of recombination, and the recombination strains CH-SCMY-2-2019-2019 and GDDX-2018-2018, both of which had major and minor parental strains, belonged to lineages 1 and 8, respectively. The analysis of the ML evolutionary tree, which aligns well with real-world data, suggests a close relationship between lineages 1, 3, and 8. Specifically, lineages 3 and 8 exhibit a tendency towards recombination mutations, confirming the predictions made by RDP4. Recombination is a reason why PRRSV continues to evolve and adapt to the environment, and its high prevalence is one of its characteristics. In recent years, strains born as a result of continuous recombination have greatly reduced the effectiveness of existing vaccines, which makes the prevention and control of blue-ear disease more challenging. Presently, in China, the predominant strains are NADC30-like and NADC34-like, followed by the HP-PRRSV strains. There are three distinct stages in the epidemiological timeline of PRRS in China, each of which includes representative strains and clinical symptoms. The first phase was from 1996 to 2006, during which the dominant epidemic strain was the C-PRRSV strain represented by the classical strain CH-1a, which was the earliest strain to be identified in China and may have led to the termination of pregnancy in sows and respiratory illnesses in swine bars (Zhao et al., 2022). In the second phase, from 2006 to 2012, HP-PRRSV strains replaced the C-PRRSV strains as the new dominant strains in China (Tian et al., 2007), which caused higher morbidity and mortality rates, more severe clinical manifestations, and affected fattening swine and gilts of all ages, adversely impacting the Chinese economic swine industry. In the final phase, from 2012 to the present, the prevalence of lineage 1 strains increased, rapidly spreading to all parts of the country, forming an independent branch and gradually becoming the new dominant prevalent spectrum in China, represented by the NADC30-like strain (Liu et al., 2021). Furthermore, the management and prevention of PRRS have become more complex owing to the ineffectiveness of existing market vaccines in hindering recombinant strains. In 2017, the intermediate virulence strain NADC34-like was widely prevalent, with clinical manifestations of reproductive disorders in sows, and the prevalence of the NADC34-like strain has increased significantly since 2020, and it may become the dominant prevalent strain in the future (Zhao et al., 2022), further complicating the clinical prevention and treatment of PRRS more difficult in China.

Our research focused on examining the genetic diversity and genetic mixing of 618 PRRSV-2 *GP3* genes across China, spanning the years 1996 to 2023, and investigated the similarities and differences in nucleotides and amino acids among various spectrum strains, PRRSV generates new dominant prevalent strains to adapt to the changes in the transmission environment through continuous mutations. Existing vaccines are unable to effectively prevent PRRS infection and transmission, which poses significant challenges to prevention and control efforts. Although the *GP3* gene exhibits relatively low conservation, it is presently considered moderately conserved. However, the potential for mutations and recombination events during future transmissions should not be overlooked. Through the analysis of *GP3* genes from diverse strains and over different years, our objective is to provide a theoretical framework and roadmap for developing novel vaccines and treatments. This research aims to mitigate the impact of porcine PRRS on the pig farming industry.

## 5 Conclusion

Between 1996 and 2023, the prevalent PRRSV-2 strains in China have been categorized into lineages 1, 3, 5, and 8. Notably, lineages 1 and 8 have shown extensive distribution, prolonged prevalence, high recombination rates, and increased mutation probabilities. These lineages are also considered the predominant strains among the commonly found variants in China. GP3 is not well conserved and is vulnerable to mutations. It plays a role in viral infection and antibody production, making it a promising research focus for developing new vaccines. By studying the gene sequence of GP3, it is possible to better understand its prevalence and mutation trends in China and screen for key mutation sites, as well as to guide the clinical selection of effective vaccine strains close to the clinic for the prevention and control of PRRS, to reduce all kinds of losses caused by PRRS.

## Data availability statement

The datasets presented in this study can be found in online repositories. The names of the repository/repositories and accession number(s) can be found in the article/Supplementary material.

## Author contributions

CL: Conceptualization, Data curation, Formal analysis, Methodology, Writing – original draft, Writing – review & editing. YZ: Data curation, Formal analysis, Methodology, Writing – original draft. KL: Data curation, Writing – original draft. GL: Conceptualization, Data curation, Formal analysis, Methodology, Writing – original draft. QL: Writing – review & editing. HZ: Writing – review & editing. HS: Writing – review & editing. RW: Writing – review & editing. WK: Writing – review & editing. MZ: Funding acquisition, Investigation, Project administration, Supervision, Writing – review & editing.

## Footnotes

1
www.ncbi.nlm.nih.gov

2
https://itol.embl.de
